# Potential of continuous cover forestry on drained peatlands to increase the carbon sink in Finland

**DOI:** 10.1038/s41598-023-42315-7

**Published:** 2023-09-27

**Authors:** Aleksi Lehtonen, Kyle Eyvindson, Kari Härkönen, Kersti Leppä, Aura Salmivaara, Mikko Peltoniemi, Olli Salminen, Sakari Sarkkola, Samuli Launiainen, Paavo Ojanen, Minna Räty, Raisa Mäkipää

**Affiliations:** 1 Natural Resources Institute Finland, Latokartanonkaari 9, 00790 Helsinki, Finland; 2 Faculty of Environmental Sciences and Natural Resource Management, Norwegian University of Life Sciences NMBU, P.O. Box 5003, 1433 Ås, Norway

**Keywords:** Plant sciences, Biogeochemistry, Environmental sciences

## Abstract

Land-based mitigation measures are needed to achieve climate targets. One option is the mitigation of currently high greenhouse gas (GHG) emissions of nutrient-rich drained peatland forest soils. Continuous cover forestry (CCF) has been proposed as a measure to manage this GHG emission source; however, its emission reduction potential and impact on timber production at regional and national scales have not been quantified. To quantify the potential emission reduction, we simulated four management scenarios for Finnish forests: (i) The replacement of clear-cutting by selection harvesting on nutrient-rich drained peatlands (CCF) and (ii) the current forest management regime (BAU), and both at two harvest levels, namely (i) the mean annual harvesting (2016–2018) and (ii) the maximum sustainable yield. The simulations were conducted at the stand scale with a forest simulator (MELA) coupled with a hydrological model (SpaFHy), soil C model (Yasso07) and empirical GHG exchange models. Simulations showed that the management scenario that avoided clear-cutting on nutrient-rich drained peatlands (i.e. CCF) produced approximately 1 Tg CO_2_ eq. higher carbon sinks annually compared with BAU at equal harvest level for Finland. This emission reduction can be attributed to the maintenance of a higher biomass sink and to the mitigation of soil emissions from nutrient-rich drained peatland sites.

## Introduction

Peatlands are some of the largest carbon reserves globally and store approximately 85% of the carbon (~ 550 Gt) of the Northern Hemisphere^1^. Approximately 12% of peatlands have been drained for a variety of land-use purposes. Peatland drainage induces aerobic decomposition, which is a significant source of CO_2_ into the atmosphere, particularly in Finland, where more than half of the peatland area (> 5 mill. ha) has been drained—mainly for forestry^2^. Avoiding peatland loss and degradation has an emission reduction potential of 813 Tg CO_2_ eq. in freshwater wetlands at the global level^3^, translating into annual peatland emission reduction by 8.35 Tg CO_2_ eq. in Finland (peat extent based on the International Mire Conservation Group Global Peatland Database^4^). According to the national greenhouse gas (GHG) inventory^5^, the CO_2_ emissions of drained peatland forest soils were 8.2 Tg in Finland for 2021, with an uncertainty of 83% (twice the relative standard error). Implementing measures that halt the degradation of peat soil and peatland vegetation would reduce these emissions. Global studies on the land-based mitigation of climate change^3^ report substantial uncertainties for their estimates, and therefore, national studies quantifying emission reduction potentials with bottom-up data and modelling are needed. National bottom-up studies are valuable also because drained peatlands are unevenly distributed across Finland, with most of them being in the western and northern regions^6^. Therefore, optimal land-based mitigation measures vary across regions, e.g., due to the extent of the drained peatland, their soil type, forest structure, regional wood demand and climatic conditions.

In Finland, forestry on peatlands has a considerable economic and ecological importance, because about a 20% of the productive forestry land i.e., about 4.1 mill ha is in drained peatlands^7^. The current practice in peatland forestry is largely based on rotation forestry, where tree stands are managed by thinning from below, the water table is kept at a low level by ditch network maintenance treatments, and after clear cut, the soil surface prepared by tillage such as mounding with furrow ploughing, and thereafter planted with seedlings.

On nutrient-rich drained peatland sites, continuous cover forestry (CCF) is a promising option as there are environmental and economic benefits, such as improved water quality and the avoidance of the costs of stand regeneration and ditch maintenance^8,9^. CCF on drained peatlands has been proposed as a measure to reduce soil GHG emissions through managing the water table level^10–12^. The rationale for using CCF on drained peatlands builds on the fact that a continuous and managed forest cover maintains the water table at the level which allows trees to grow, further ditch-network maintenance is not needed, and both nutrient loading to waterways and GHG emissions are reduced^9^. The reduction of soil GHG emissions with CCF on drained peatlands assumes that the mean water table level (WTL) is higher, reducing soil CO_2_ emissions compared with even-aged management. Site level studies and models show that peat hydraulic conductivity, ditch spacing, ditch depth and vegetation evapotranspiration affect WTL and the thickness of the fast decomposing aerobic peat layer^13,14^.

Nutrient-rich drained peatlands are typically occupied by Norway spruce-dominated forests, and they are the most productive site types of the boreal forests^2^. In Finland, most of these sites have been drained for forestry more than 50 years ago and are currently in the late stage of drainage succession, where the mire plant species have been replaced by upland forest vegetation consisting of mesic herb and forest moss species and where bilberry (*Vaccinium myrtillus*) is the most frequent dwarf shrub. The age structure of drained peatland forests is such, that a large fraction of those are currently at mature stage and approaching decision whether those will be clear-cut or managed with selection harvests.

Studies estimating the climate benefits of CCF management on drained peatlands are few and focused on stand level assessments^15,16^. Stand level climate change mitigation studies exclude potential leakage effects where e.g., reduced loggings in a study site are taking place elsewhere, and therefore, larger-scale simulation studies with alternative forest management strategies are needed. At a stand level, CCF harvesting typically removes between 50 and 70% of the merchantable timber, while clear-cut harvesting removes nearly 100% of the biomass, and in Southern Finland a mature nutrient-rich drained peatland sites may have 250–400 m^3^ in stem volume per ha. The application of CCF has been limited partly due to limited evidence based on tree growth and productivity compared with that of even-aged rotation forestry, especially with suppressed Norway spruce trees after selection harvests. On a peatland site in Canada, the release effect on the diameter growth of suppressed Black spruce (*Picea mariana* (Mill.)) trees took 12 years^17^. On a drained peatland site (Lettosuo) in southern Finland, it took less than 5 years for suppressed Norway spruces to recover their diameter growth after selection harvest; however, the carbon uptake by trees was increased immediately after the harvest treatment^18^.

To develop climate policies and incentives for land-based climate change mitigation, countries should be able to quantify the technical and feasible potential of different climate change mitigation measures. Currently, the national level scenario studies focusing on land-based mitigation strategies in forests are few and mostly consider the impact of harvest levels or climate change on forest carbon sinks^19,20^. In a market-driven economy, reducing harvests as a mitigation measure can be applied on state lands, but on private lands, this is more challenging without additional policy instruments. Therefore, there is an urgent need for alternative forest management strategies that allow timber provisioning while having simultaneous environmental benefits. Previous scenario studies have shown the impacts of harvesting on forest carbon sinks^19–21^, but there are a few studies that quantify the potential impact of a management regime change (from rotation forestry to CCF) to national-level climate benefits.

To explore the potential of land-based mitigation strategies with forests, we simulated different scenarios for Finnish forests with the MELA forest simulator for 2016–2050, the SpaFHy-peat hydrological model, the Yasso07 soil carbon model and GHG exchange models (see “Methods”). We quantified the GHG exchange of tree biomass and soils for Finnish forests (both mineral soils and drained peatlands) under current (BAU) management regime and with the compulsory avoidance of clear-cut on nutrient-rich drained peatland forests (CCF). We assessed the impacts of these scenarios following two harvest levels, actual harvests (AH, which was mean annual harvesting level of the years 2016–2018), and the maximum sustained yield (MSY), which was substantially larger (7–17 mill m^3^ per year) harvest level compared with AH. The period of 2016–2018 for AH logging level is also a basis for the land-use sector targets for EU member states for 2030 (EU regulation 2018/841). The MSY logging level is the basis of Finnish forest policy by providing level of loggings that are considered feasible without compromising future logging possibilities. This allows policy-makers and the forest industry to consider timber industry development. The MSY logging level integrates current conservation statuses, however ignores possible future policy shifts, such as shifts required to meet climate targets or coming requirements of EU restoration act.

Specifically, we focused on the emissions and sink estimation of drained peatlands by incorporating the latest research findings on CO_2_, N_2_O and CH_4_ dynamics and the impact of hydrology to emissions into our simulations. In addition to BAU and CCF, we simulated a CCF-regressed (CCF-reg) scenario to study the sensitivity of our results on the growth level of suppressed Norway spruces after selection harvest. The compulsory avoidance of clear-cuts (CCF scenario) was based on studies that have shown a reduction in soil GHG emissions from drained fertile peatlands after the water table level had been increased^11,12^.

Our aims were as follows: (i) to quantify the forest GHG exchange differences between the current management regime (BAU), targeted peatland CCF, and increased harvest levels in Finland, (ii) to quantify CO_2_, N_2_O and CH_4_ exchange estimates for drained peatland forest soils and (iii) to identify the regions in Finland that have climate change mitigation potential with their drained peatlands.

## Results

**Figure 1:**
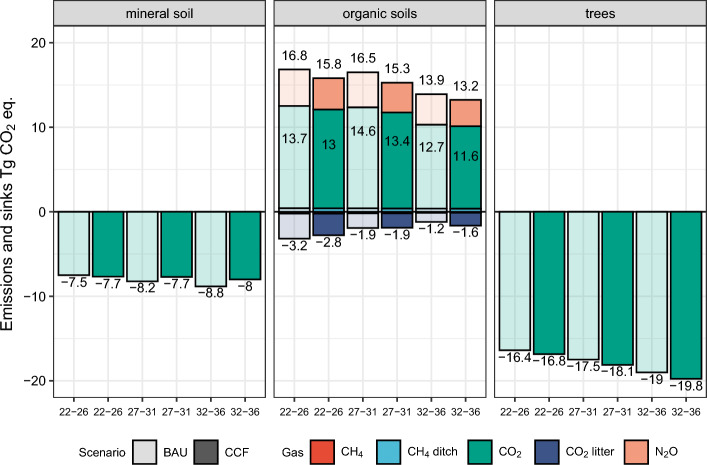
GHG exchange [Tg CO_2_ eq.] for Finnish forests according to components (trees, mineral soils and organic soils) in BAU and CCF, based on actual harvesting levels for 2022–26, 2027–31 and 2032–36. Forest management scenarios were business as usual (BAU) and the management regime where clear-cutting was avoided in the nutrient-rich peatland forests (CCF). Negative values are net carbon sinks, and positive values are net emissions (sources). Darker colours indicate sinks and emissions for the CCF scenario while lighter colours indicate sinks and emissions for the BAU scenario. Values next to the bars indicate net emissions and sinks, whereas for organic soils, values above and below the bars are gross emissions and gross sinks, respectively.

Our results indicate that by avoiding clear-cutting through the use of selection harvesting (CCF scenario) on nutrient-rich drained peatlands, it is possible to obtain climate benefits, i.e., higher vegetation carbon sinks and lower soil GHG emissions, compared with business as usual (BAU) forest management (Fig. 1). With actual harvesting (AH) levels (Table 1), the CCF scenario produced 0.7–1.3 Tg CO_2_ eq. year^−1^ higher carbon sink compared with BAU throughout the simulation period (Table 2). This difference between the CCF and the BAU scenario was mainly due to larger areas of clear-cuts in the BAU scenario and emissions related to those, as contrary to our expectations water table levels were equal between BAU and CCF or deeper for the CCF (see Supplementary Fig. S3). Our simulations for the first 10-year period showed that under the CCF scenario, the annual clear-cut area in Finland was 61,000 ha on nutrient-poor peatlands (168,000 ha mineral soils), whereas under the BAU scenario, it was 75,000 ha on all peatlands (157,000 ha in mineral soils). The total amount of harvested wood from drained nutrient-rich peatland forests was 6.7 mill. m^3^ under CCF and 9.4 mill. m^3^ under BAU for the first 10-year period, due to leakage of harvesting from peatlands to mineral soils in the CCF scenario. With the actual harvesting levels (AH), the total annual harvesting area for commercial timber was 466,300 ha for the BAU scenario and 481,700 ha for the CCF scenario in the first 10-year period.

**Table 1:** Annual increments (i.e. tree growth) and annual harvesting levels [million m^3^ year^−1^] of Finnish forests for the first three decades as simulated by MELA, according to different harvesting levels and scenarios. Actual harvesting levels are highlighted in grey. The harvest amount have been presented separately also for nutrient-rich drained peatlands (where under CCF scenario clear-cuts were not allowed). (i) The business as usual (BAU) scenario mimics rotation forestry with thinning and clear-cutting treatments, which is the most common regime in Finnish forestry. (ii) The alternative management scenario (CCF) is based on the BAU scenarios but with the additional constraint that the nutrient-rich drained peatland forests will only be managed with selection harvest practices. (iii) CFF-regressed (CCF-reg) is a scenario with decreased growth of previously suppressed Norway spruces. This scenario is a modification of the CCF scenario by reducing the growth of suppressed Norway spruce by 25% during the first 5-year period after the selection harvest.

Harvestings	Scenario	Data	Million m^3^ year^−1^		
			2016–2025	2026–2035	2036–2045
Maximum sustained yield	BAU	Increment	105.8	104.3	104.2
Maximum sustained yield	CCF	Increment	106.2	105.6	106.0
Maximum sustained yield	CCF-reg	Increment	105.1	104.0	104.2
Maximum sustained yield	BAU	Harvesting total	80.5	88.9	89.5
Maximum sustained yield	CCF	Harvesting total	78.3	87.1	87.9
Maximum sustained yield	CCF-reg	Harvesting total	76.8	85.8	86.8
Maximum sustained yield	BAU	Harvesting on nutrient-rich peatlands	9.3	10.5	8.5
Maximum sustained yield	CCF	Harvesting on nutrient-rich peatlands	6.8	8.1	7.4
Maximum sustained yield	CCF-reg	Harvesting on nutrient-rich peatlands	6.6	7.8	7.1
Actual harvestings	BAU	Increment	105.5	106.1	109.7
Actual harvestings	CCF	Increment	105.6	106.3	110.2
Actual harvestings	CCF-reg	Increment	104.1	104.1	107.5
Actual harvestings	BAU	Harvesting total	73.0	72.9	73.0
Actual harvestings	CCF	Harvesting total	72.9	72.8	72.8
Actual harvestings	CCF-reg	Harvesting total	72.9	72.8	72.9
Actual harvestings	BAU	Harvesting on nutrient-rich peatlands	9.4	7.7	6.5
Actual harvestings	CCF	Harvesting on nutrient-rich peatlands	6.7	6.2	5.8
Actual harvestings	CCF-reg	Harvesting on nutrient-rich peatlands	6.7	6.2	5.6

**Table 2:** The GHG sinks (negative values) and emissions (positive values) [Tg CO_2_ eq. year^−1^] of Finnish forests in CCF and BAU scenarios at actual harvesting (AH) and maximum sustained yield (MSY) harvesting levels.

Harvestings	Scenario		The GHG sinks and emissions [Tg CO_2_ eq. year^−1^]		
			2022–2027	2028–2037	2038–2047
AH	BAU	Tree biomass	− 16.38	− 18.57	− 22.56
AH	CCF	Tree biomass	− 16.85	− 19.28	− 23.3
MSY	BAU	Tree biomass	− 4.33	2.42	2.41
MSY	CCF	Tree biomass	− 8.31	− 1.79	− 1.8
AH	BAU	Organic soils	13.53	13.6	13
AH	CCF	Organic soils	12.91	12.42	12.32
MSY	BAU	Organic soils	12.03	12.48	12.45
MSY	CCF	Organic soils	11.67	11.32	11.26
AH	BAU	Mineral soils	− 7.35	− 8.64	− 12.15
AH	CCF	Mineral soils	− 7.53	− 7.91	− 11.43
MSY	BAU	Mineral soils	− 3.01	− 4.23	− 6.73
MSY	CCF	Mineral soils	− 2.96	− 4.08	− 6.48
AH	BAU	Total	− 10.2	− 13.61	− 21.72
AH	CCF	Total	− 11.46	− 14.77	− 22.41
MSY	BAU	Total	4.69	10.68	8.13
MSY	CCF	Total	0.41	5.46	2.98

With the maximum sustained yield (MSY), the total annual harvesting amount was approximately 3 mill. m^3^ lower under the CCF management scenario compared with the BAU scenario (Table 1). This difference was due to fact that clear-cuts were not allowed on the nutrient-rich drained peatlands under the CCF scenario. For the period of 2022–2027, the Finnish forests were emission sources, according to the MSY harvesting level, with BAU management resulting in emissions that were 4.2 Tg CO_2_ year^−1^ eq. higher than those under the CCF scenario (Table 2).

**Figure 2:**
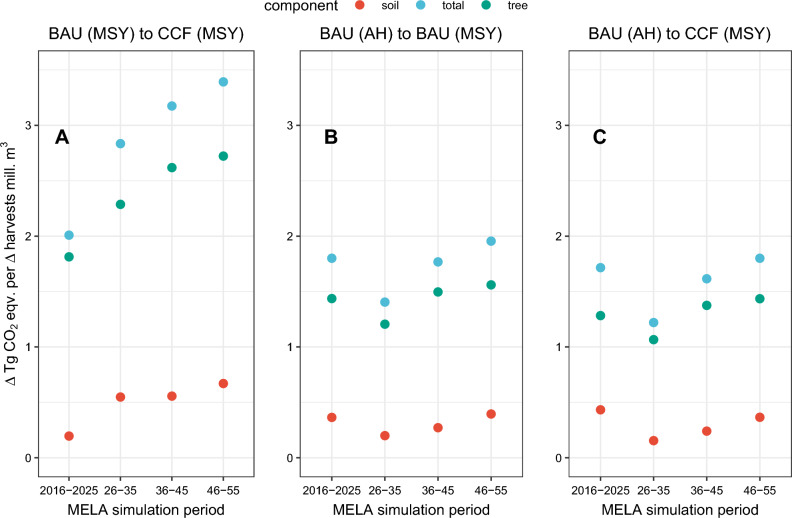
Ratio between change in the GHG exchange [Tg CO_2_ eq.] of forests relative to the change in the harvest removal [mill. m^3^] for different periods for soils, trees biomass and total ecosystems for Finland. The left panel (**A**) illustrates the ratio when BAU management is converted to CCF management under the MSY harvesting level. The middle panel (**B**) illustrates the ratio when converting from AH harvesting levels with BAU management to MSY harvesting level with BAU. The right panel (**C**) illustrates the ratio when converting from AH harvesting levels with BAU management to MSY harvesting level with CCF. Note that the comparison of the ratio between change in GHG exchange and change in harvest removals was not meaningful for the transition from BAU (AH) to CCF (AH) as denominator (change on harvest) is close to zero.

According to our results, the relative climate impact per change in harvests (ΔTg CO_2_ eq. per Δ harvested mill. m^3^) were smaller when converting from BAU (AH) to CCF (MSY) management on nutrient-rich peatlands compared with conversion from BAU (AH) to BAU (MSY) (Fig. 2). Increasing harvests from the current management (BAU) and AH logging levels to MSY harvesting level but with CCF management increased less CO_2_ emissions than conversion from BAU (AH) to BAU (MSY). Reducing harvesting at the MSY harvesting level by switching from BAU (80.5 mill. m^3^) to CCF (78.3 mill. m^3^) produced 2–3.5 Tg CO_2_ eq. climate benefits per 1 mill. m^3^, whereas that ratio was 1.3–2 Tg CO_2_ eq. per 1 mill. m^3^ when the harvesting level changed between the AH level (73 mill. m^3^) and the MSY harvesting level (80.5 mill. m^3^) under the BAU management (Fig. 2). Most of the climate benefits due to reducing harvests with the transition from BAU (MSY) to CCF (MSY) were obtained by the increased tree biomass sink. Note that these estimates exclude carbon sinks with harvested forest product pool and potential substitution benefits.

**Figure 3:**
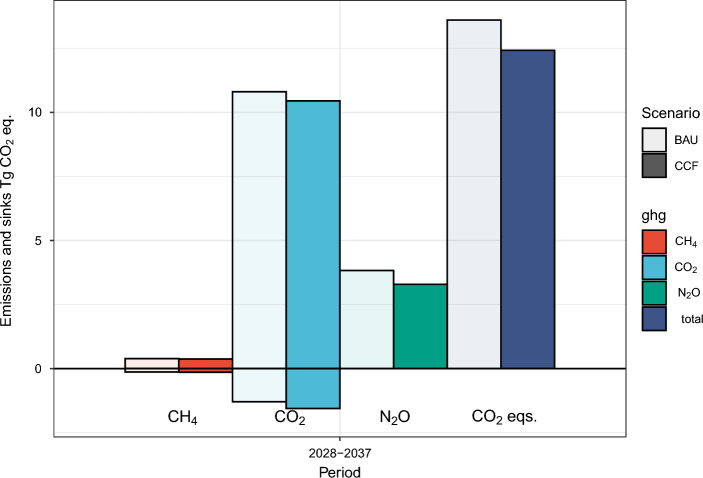
Average annual soil GHG emissions and sinks [Tg CO_2_ eq.] from drained peatland forest soils for 2028–2037 under BAU and CCF management scenarios, assuming that the actual harvesting level is maintained in Finland throughout the simulation period. The CH_4_ describes the methane emissions from ditches and methane sinks on the peat surface. The CO_2_ describes emissions (decomposing peat) and sinks (accumulation of organic matter originating from natural mortality and harvest residues). N_2_O describes emissions from the peat surface, while CO_2_ eqs. on right is the sum of CH_4_, CO_2_ and N_2_O based IPCC AR4 GWP ratios.

At actual harvest levels, the net emissions from drained peat soils were 14.5 Tg CO_2_ eq. year^−1^ for 2022–2035 in the BAU scenario and approximately 13.5 Tg CO_2_ eq. year^−1^for the CCF scenario (Table 3). The soil emissions reductions decreased in periods close to 2040s (Figs. 1, 3). The largest individual component of these emissions was the CO_2_ emission from the peat layer, being approximately 12 Tg CO_2_ year^−1^ before 2035 for the BAU scenario. The second largest component was the N_2_O emission with approximately 4.1 Tg CO_2_ eq. year^−1^ before 2035 for the BAU scenario (Table 3). Both CO_2_ and N_2_O emissions were higher than those reported earlier as we also accounted for the additional emissions from the clear-cut area^22–24^.

**Table 3:** The GHG exchange [Tg CO_2_ eq.] from drained peatland sites in the CCF and BAU scenarios at actual harvesting levels for Finland for 2022–2035 and 2022–2050. CH_4_ refers to methane sinks and emissions from peat soil surface, and CH_4_ ditch refers to methane emissions from ditches. CO_2_ refers to emissions from peat at the soil surface. CO_2_ litter refers to sinks and emissions from decaying large wood litter and harvest residues, and N_2_O refers to emissions from peat and litter at the soil surface.

Gas	Scenario	GHG exchange Tg CO_2_ eq.	
		2022–2035	2022–2050
CH_4_	BAU	− 0.19	− 0.09
CH_4_	CCF	− 0.2	− 0.08
CH_4_ ditch	BAU	0.41	0.39
CH_4_ ditch	CCF	0.41	0.38
CO_2_	BAU	12.09	11.17
CO_2_	CCF	11.52	10.69
CO_2_ litter	BAU	− 1.91	− 1.59
CO_2_ litter	CCF	− 1.81	− 1.44
N_2_O	BAU	4.13	3.87
N_2_O	CCF	3.55	3.34
Total	BAU	14.53	13.75
Total	CCF	13.47	12.89
Difference	BAU-CCF	1.06	0.86

**Figure 4:**
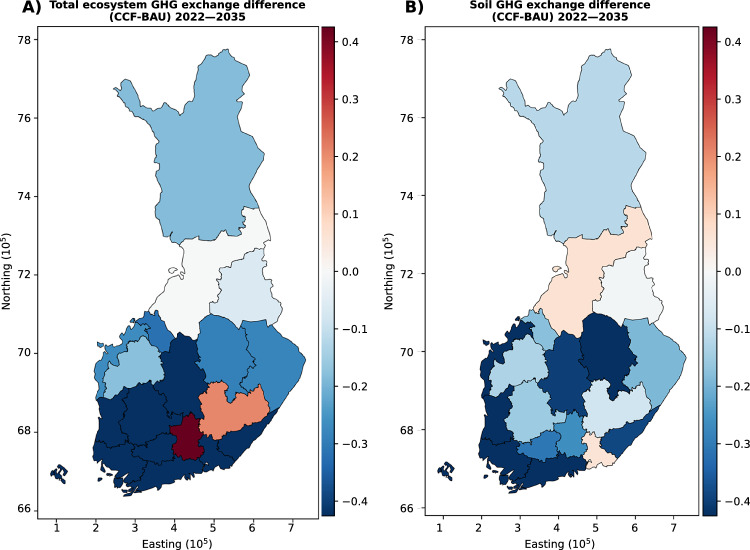
Differences in GHG exchange [Gg CO_2_ eq. per ha of forest] of regions between the CCF and BAU scenario at actual harvesting levels for 2022–2035. Panel (**A**) presents the total GHG exchange for ecosystems, while panels (**B**) presents the soil GHG exchange. Positive values indicate that the CCF scenario has a less favorable impact on GHG emissions than the BAU scenario. Base map: National Land Survey Finland (https://www.maanmittauslaitos.fi/en) with map projection: ETRS89.

Most of the regions showed reduced emissions for the CCF scenario compared with the BAU scenario with total ecosystem GHG exchange for the period of 2022–2035 (Fig. 4) and for 2022–2050, with identical harvesting levels (actual harvestings, Fig. 4 and Supplementary Table S9). These emission reductions can be attributed to the regions that have significant areas of nutrient-rich drained peatlands and areas that have high harvesting possibilities during these periods^25^ on these sites (Supplementary Tables S6–S9), such as Central Finland, Lapland, Northern Ostrobothnia and North Karelia (see also Supplementary Material for more details).

Changing the management regime from BAU to CCF resulted in a leakage effect, where more harvestings targeted the mineral soils in the CCF scenario compared to the BAU scenario. The magnitude of the leakage for the mineral soil carbon stock change was between − 0.3 and − 0.9 Mt CO_2_ over different simulation periods with AH levels (Table 4). It should be noted that in spite of the leakage, the CCF scenario resulted in 0.2–1.3 Tg CO_2_ eq. year^−1^ higher carbon sinks for Finnish forests compared with the BAU scenario.

**Table 4:** Differences in total GHG exchange between CCF and BAU scenarios [Tg CO_2_ eq.] for Finland by components as in the GHG inventory (mineral soils, organic soils and tree biomass). Differences were estimated by deducting BAU sinks/emissions from those of CCF. Positive values for mineral soils indicate a leakage effect due to more extensive harvestings on mineral soil sites for CCF compared with BAU. Negative values in the table indicate that CCF provides larger climate change mitigation benefits compared to BAU. Actual harvesting levels were used.

Component	The difference between CCF and BAU in Tg CO_2_ eq.					
	2022–2026	2027–2031	2032–2036	2037–2041	2042–2046	2047–2051
Mineral soils	− 0.17	0.53	0.82	0.91	0.64	0.28
Organic soils (peatlands)	− 0.45	− 1.20	− 1.10	− 1.39	− 0.18	− 0.04
Trees	− 0.47	− 0.62	− 0.76	− 0.82	− 0.71	− 0.40
Total	− 1.09	− 1.29	− 1.03	− 1.31	− 0.25	− 0.15

Based on the results of the sensitivity analysis (CCF-reg scenario), which assumed a reduced growth of suppressed Norway spruce trees after selection harvests (− 25% over next 5 years) reduced the carbon sink in Finland compared with BAU and CCF scenario (Supplementary Table S10 in the Supplementary Material). According to the CCF-reg scenario ran with the AH levels, the total carbon sink of Finnish forests was 1–3 Tg CO_2_ year^−1^ eq. lower than that under the BAU scenario.

## Discussion

### GHG exchange of scenarios

We found that the CCF scenario provides approximately 1 Tg CO_2_ eq. higher carbon sinks for Finnish forests before 2035 compared with the BAU scenario, which can be attributed to the avoidance of clear-cuttings, resulting in reduced soil emissions and stronger carbon sinks in tree biomass. The role of clear-cut emissions is significant, especially as the interplay between reduced ditch depth and increased LAI resulted, that there were more deeper water table levels with CCF compared to BAU with drained peatlands. Avoiding clear-cuttings under the CCF scenario resulted in an increase in the total annual harvesting area of commercial timber by 15,000 ha, with a more pronounced harvesting of mineral soil areas. This leakage to mineral soils can be seen in the results, where the sink of mineral soils was reduced by 0.3–0.9 Tg CO_2_ eq. from 2027 onwards under the CCF scenario compared with that under the BAU scenario with AH. Despite this reduction of the sink due to leakage however, the CCF scenario produced higher overall carbon sinks for Finland than the BAU scenario. This result shows that there are opportunities to maintain timber production and simultaneously reduce GHG emissions with climate-smart forest management planning.

### Relative climate impacts of different scenarios with varying harvest amounts

The relative climate impact (the change in GHG sink relative to the change in the harvesting amount) doubled when reducing harvestings by converting from BAU (MSY) to CCF (MSY) compared with reducing harvestings while retaining BAU management [i.e. from BAU (MSY) to BAU (AH)]. Also, it was found that increasing harvests from the AH level to MSY level, emissions increased less per 1 mill m^3^ if the management was converted to CCF instead staying with current forest management (BAU). Most of these climate benefits were due to stronger tree biomass sink. The soil-related climate benefits were larger in the case of a management regime change from BAU to CCF compared with changes in the harvesting levels with current management (BAU) regime due to fact that conversions from BAU to CCF reduces the amount of stands with recent clear-cuts and high soil related emissions.

Our results also show that these relative climate impacts, measured as a ratio between the change in GHG sink relative to the change in the harvesting amount is not a constant and varies over time; its dynamics differ between conversions from current management regime (BAU) to CCF compared with maintained management regime (BAU), but changed harvests.

The varying relative climate impacts with time suggests that climate benefits will increase over time for CCF compared with BAU. Earlier studies have shown that these ratios vary from 1.2 to 1.8 (depending on the models used), when the unit was the change in the total forest sink in CO_2_ compared with that change in the harvested stem biomass as CO_2_ (excluding emissions from drained peatland soils)^26^. According to a review^27^, the ratio between forest carbon stock change and change in the harvesting varied over time and across regions. Increasing harvestings by 1 unit of C would decrease the forest carbon sink by 1.6 units of C on average^27^ (with high variability and a standard deviation of 0.8–1.1, based on the statistical cut-off). This is supported by our results regarding the case, where staying with current management, where the ratio between changes in tree biomass sink and in harvests varied between 1.2 and 1.6. The ratio varied between 1.8 and 2.7 with management change from the current management to CCF; the unit is Δ Tg CO_2_ eq. per Δ harvested mill. m^3^. These values convert to 1.45–1.94 (staying with current management) and to 2.18–3.27 (from current management to CCF), respectively, unit being as change in carbon sink relative to change in harvested stem biomass in carbon, assuming a biomass expansion factor of 0.45 Mg m^−3^ and a carbon content of 50%^28^.

Our results show that for tree biomass, the relative climate impact, as a ratio between changes in sink and harvesting amount reported in this study is much larger than those reported earlier^27^ when harvesting reduction is a result of management practice change (current management to CCF). Likely, the reason for this relates to the fact that the conversion from current management to CCF results in a larger area managed with lower intensity by selection harvests and in a smaller total clear-cut area, leading to a smaller area of young seedling stands with negligible volume growth and marginal tree biomass carbon accumulation.

### The impact of clear-cut emissions

The inclusion of additional clear-cut emissions of CO_2_ and N_2_O based on^22–24^ resulted in higher GHG emissions from drained peatland soils at national level than those reported under the GHG inventory^6^. Our estimates for CO_2_ emissions as a result of peat and litter decomposition varied between 9.2 and 10.2 Tg CO_2_, depending on the period, whereas the reported emissions for Finland in the year 2021 were 8.2 Tg CO_2_^5^. This difference can be mainly attributed to the emissions from clear-cut areas as the annual CO_2_ emissions during the first 5 years after clear-cut on nutrient-rich drained peatlands vary between 8 and 31 Mg CO_2_ per ha, whereas the total annual clear-cut area on drained peatlands reached 30,000 ha per year in the BAU scenario. Also, the contribution of the N_2_O emissions to the total GHG exchange of drained peatlands was larger in our study compared with that of the GHG inventory^29^, where N_2_O emission estimates are solely based on data from tree-covered study sites, excluding those that have been recently clear-cut^30^.

### Regional results on the GHG exchange

Our regional results on the climate benefits of converting from BAU to CCF in nutrient-rich peatlands can be roughly divided into three categories: (i) regions with no significant differences, (ii) regions where benefits were found with tree biomass and (iii) regions where benefits were found with tree biomass and organic soils. Typically, regions where climate benefits were not found were those without significant areas of nutrient-rich drained peatland sites, whereas the regions where benefits were observed, especially regarding tree biomass, were those with high recent harvesting levels (e.g., in 2016–2018) (actual harvestings larger than the maximum sustained yield). Results of this work could be utilised when disseminating results, planning incentives, streamlining exiting policy instruments and when prioritising regions for conversion from current management to CCF of nutrient-rich drained peatlands.

### Future development needs for the scenario modelling of CCF

Our modelling framework has been built on various published models that have been here linked together. However, a clear drawback with our approach is that the forest simulator MELA lacks the feedback from soil conditions (beyond static forest site fertility type), weather variability (beyond annual temperature sum and site location) and understorey vegetation competition to growth, which means that, e.g., dynamic nutrient availability, elevated peatland water table and droughts on mineral soils are not reflected in the simulated tree increment. Overcoming these shortcomings should be a future research priority. For tree increment we used empirical growth models of MELA for peatlands to describe the development of the stands after selection harvestings. To evaluate the potential of reduced growth from selection harvestings and the impact of growth models, we conducted a sensitivity analysis (CCF-reg scenario) where we downscaled the growth of suppressed Norway spruce trees by 25% for 5 years after selection harvesting. This resulted in the loss of climate benefits that were shown by the CCF scenario compared by the BAU scenario. The assumed 25% growth reduction for 5 post-harvest years is highly uncertain and mimics the potential release effect of diameter growth, which typically occurs as a delay after management treatment; however, it is likely an overestimation of the reduction in carbon uptake after selection harvest^18^.

### Uncertainty related to GHG exchange of the scenarios

According to the GHG inventory of Finland, the total uncertainty for the GHG exchange of forests land was 10.6 Tg CO_2_ (83%)^29^, which can be considered as a minimum estimate for the uncertainty with model-based values presented here for Finland. According to a Monte Carlo simulation study, standard deviation of forest carbon sink in Finland was 27% for 1990–2004^31^ and it was found that soil model initialisation was the most influential parameter for total uncertainty. For mineral soils, in the GHG inventory it was reported that the propagated uncertainty (uncertainty of sampling, biomass model, litter rates and soil model parameters) of the soil carbon sink was 26–32% depending on the region in Finland^32^. For drained peatlands soil emissions uncertainties are relatively high in the GHG inventory, but still less than actual estimate showing that drained peat soils are an emissions source in Finland^6^. Our findings are based on comparisons between the BAU and CCF scenarios, and we can assume that the uncertainties of the estimates of these different scenarios are highly correlated^20^; consequently, the uncertainty in the difference between the scenarios is smaller. The major uncertainty regarding our results in addition to growth models of suppressed trees (see CCF-reg sensitivity analysis) is based on the estimation of CO_2_ and N_2_O emissions that originate after clear-cutting from drained peatland sites. To illustrate the impact of the chosen assumptions, we used a linear decrease of soil CO_2_ emissions for 8 years, instead of 10 years, showing that the difference between BAU and CCF would narrow down by 0.3 Tg CO_2_ per year if additional emissions after clear-cutting would reduce faster than assumed here (Supplementary Fig. S2 in the Supplementary Material). In contrast, using a 50% lower emission factor for N_2_O for nutrient-rich drained peatlands after clear-cutting would imply an approximately 0.6 Tg CO_2_ eq. smaller difference between BAU and CCF (Supplementary Fig. S1 in the Supplementary Material). This underlines the need for additional studies of GHG fluxes, especially N_2_O, after clear-cutting. Simultaneously, these extremely high CO_2_ and N_2_O emissions from clear-cut areas highlight the fact that even-aged forestry is questionable on these nutrient-rich drained peatlands and that alternative management strategies should be tested. We also highlight the need for a mechanistic model with mass-balance for drained peatland ecosystems, allowing the estimation of CO_2_, CH_4_ and N_2_O exchange.

There is an urgent need for land-based climate change mitigation strategies, and our results show that CCF forestry on nutrient-rich drained peatlands has potential for emission reduction without compromising wood production despite the leakage of harvestings to mineral soils.

## Methods

To estimate the future development of GHG exchange of Finnish forests, we followed the methods and system boundaries of the Finnish GHG inventory for forest land^29^ with a few exceptions. Our simulations include productive forests, where growth is more than 1 m^3^ ha^−1^, equivalent to the national forest definition, whereas in the GHG inventory, they apply a slightly broader forest definition by the FAO. The total area of Finnish forests (productive and poorly productive forests) used in this study was 22.8 mill. ha, according to the National Forest Inventory (NFI) (2014–2018), and the area of nutrient-rich drained peatlands was approximately 2 mill. ha. The regions with the largest coverage with drained peatlands were North Ostrobothnia, Lapland, Kainuu, and North Karelia (see Supplementary Tables S7 and S8).

**Figure 5:**
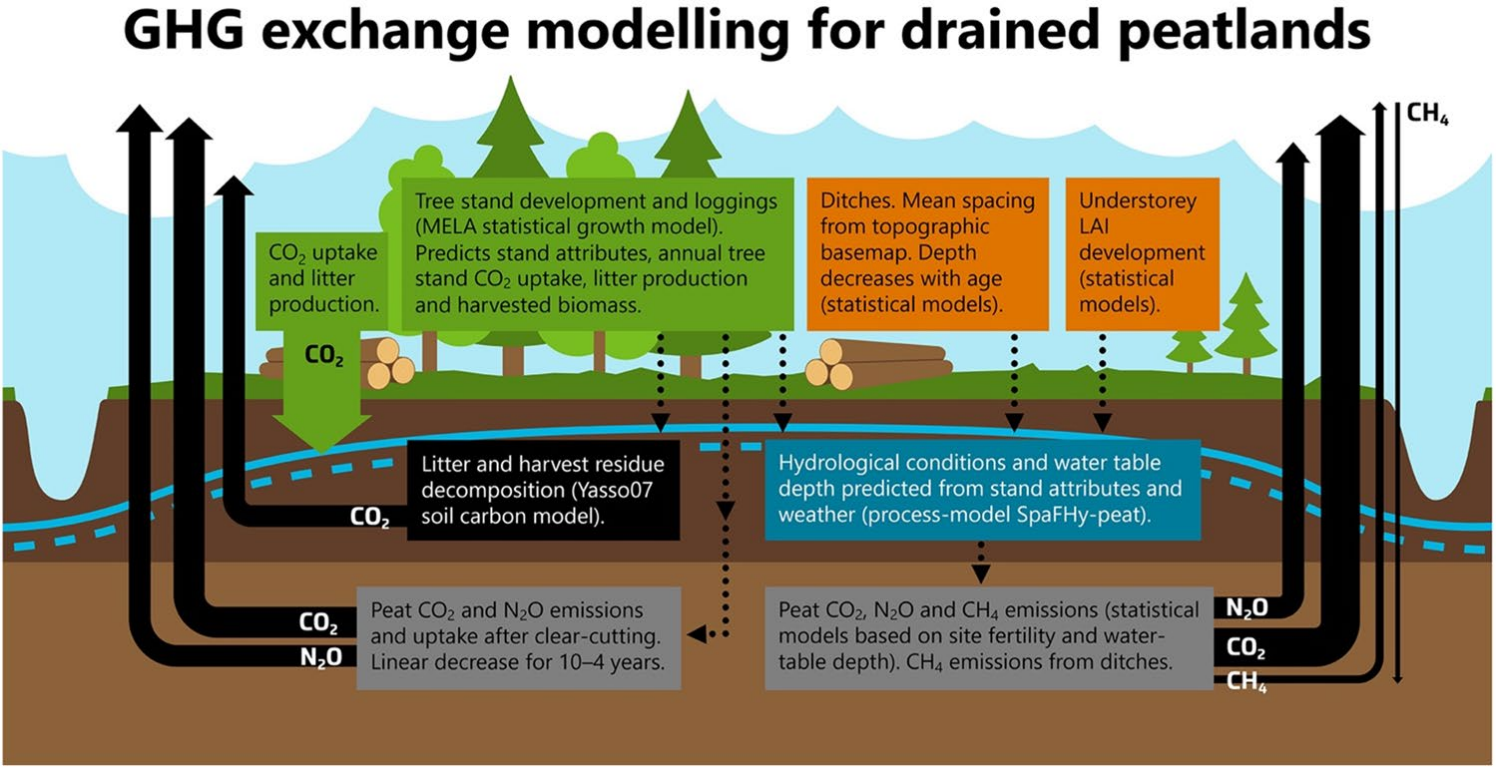
Illustration of the GHG exchange modelling for the Finnish drained peatland forests in this study by using the MELA forest simulator, the hydrological SpaFHy-peat model, the Yasso07 soil carbon model and empirical models. The green boxes indicate vegetation related modelling and element fluxes, while the orange boxes indicate drivers for the hydrological model. The black box describes modelling of organic matter decay with the Yasso07 model, and the blue box describes water table depth prediction with the SpaFHy-peat model. Grey boxes describe the empirical modelling of CO_2_, CH_4_ and N_2_O emissions for drained peatlands, for both, clear-cut areas and for tree covered stands.

Our simulations assume constant land-use, and therefore, all potential future land-use changes were excluded. The main methodological difference between our work and the GHG inventory is that here GHG exchange of drained peatland forest soils was estimated with the water table depth-driven models and with models that consider increased soil emissions after clear-cut harvesting. In addition, these estimations were conducted separately for each calculation unit based on the NFI data (stand scale) and then upscaled to regional scale, whereas in the GHG inventory, the carbon stock change and GHG exchange estimates were determined at the larger regional scale, using regional mean values. Here, CH_4_ emissions and CO_2_ sinks of undrained peatland soils were excluded, whereas drained peatland soils were included, as in the GHG inventory (Fig. 5).

The simulated projections were initiated for the year 2016 using the National Forest Inventory data (NFI12)^7^, representing forests of Finland and measured in 2014–2018 for southern Finland and in 2012–2013 for northern Finland. The total number of calculation units on forests was 57,720, and they were used with the MELA forestry model (of which 12,246 were in drained peatland forests). These calculation units were classified into three categories according to restrictions concerning wood production: (1) no restrictions for wood production, (2) restrictions for wood production and (3) no wood production allowed. This classification defines the management operations that are allowed for each calculation unit.

### MELA modelling framework and scenarios

The future development of forest resources in Finland was estimated using the MELA forestry model for 2016–2050, version MELA2016^33,34^. With MELA, we simulated a large number of management schedules for each calculation unit throughout the calculation period, based on the individual tree growth and development models^35^, forest management guidelines^36^ and estimates of the economics using costs for management actions and prices for harvested wood^37^. Different scenarios for the forests were obtained from the simulated management schedules with the linear optimisation package^33,38^ under the chosen objectives and constraints^37^. Development of forests resources was conditional on the harvesting levels that changed the wood use and tree increment, according to the simulated harvestings. The results of the MELA modelling were used to determine the GHG exchange of forests growing on mineral soils and peatlands.

In this study, we had two scenarios for the harvesting level: (i) maximum sustained yield and (ii) actual harvesting (MELA 2021). The maximum sustained yield scenario considers the long-term wood production and the economics. The harvested removal was defined by maximising the net present value of the 4% discount rate subject to non-declining periodic total roundwood and energy wood removals, saw log removals and net income (MELA 2021). With the maximum sustained scenario, the harvesting volumes ranged between 76 and 90 mill. m^3^ annually. The actual harvesting scenario was estimated by using annual harvests equal the mean level of harvests from 2016 to 2018, being 73.6 mill. m^3^.

In addition to the two scenarios for harvest level, we had two different scenarios for the applied forest management regime. The business as usual (BAU) scenario mimics rotation forestry with thinning and clear-cutting, which is the most common regime in Finnish forestry, and in this scenario, CCF management was not allowed. The alternative management scenario (CCF) is based on the BAU scenarios but with the additional constraint that the nutrient-rich drained peatland forests will only be managed without clear-cuts, i.e., they were transferred to continuous cover forestry when MELA conducted harvesting operations and when the minimum basal area thresholds were met. Nutrient-rich drained peatlands were defined as sites being of the *Myrtillus* type or herb-rich type sites, representing the most productive peatland sites for wood production, excluding Scots pine-dominated *Myrtillus* sites^39^.

To study the sensitivity of the tree growth to the results, a scenario with decreased growth of previously suppressed Norway spruce after selection harvesting was tested, termed as CFF-regressed (CCF-reg) scenario. In this scenario, it was assumed that other species than Norway spruce grew according to the default model estimates. The CCF-reg scenario is a modification of the CCF scenario by reducing the growth of suppressed Norway spruce by 25% during the first 5-year period after the selection harvest. The reduction of 25% is an expert judgement but an conservative estimate as, most likely, the reduction in growth is not greater than 25%, as described for the Lettosuo drained peatland area^18^.

The time step for MELA simulations was 10 years for tree biomass, whereas that for soil carbon and GHG exchange was 1 year. The simulation results were presented at minimum with 5-year periods, where linear interpolation between the MELA simulations for tree biomass was assumed, and then merged with the annual soil GHG exchange data.

### Implementation of selection harvests in the MELA system

Selection harvest practices were implemented into MELA by modifying thinning routines and applying selection harvesting with the CCF (and CCF-reg) scenario on those calculation units where clear-cuts were not allowed. In the selection harvest, the trees were removed also from the dominant canopy layers compared with normal thinning, where harvested trees were selected from the lower canopy layers, mainly targeting smaller trees.

Selection harvests were allowed once the stand basal area for the plot had achieved a threshold of 22 m^2^ per ha. The stand would be harvested until the basal area was taken down to 12 or 15 m^2^ per ha at minimum in southern and northern Finland, respectively, in Norway spruce-dominated stands. In Scots pine-dominated stands, the post-harvest basal area was at minimum 14 or 17 m^2^ per ha in southern and northern Finland, respectively. For sites dominated by deciduous tree species, selection harvest was allowed when the basal area was more than 16–21 m^2^, depending on the region and soil fertility. The basal area of deciduous stands after harvesting varied between 10 and 15 m^2^, depending on the region and the site’s fertility. These harvesting guidelines were applied according to the dominant species, site fertility and region, see thinning rules in Table 5 and Supplementary Fig. S2 in the Supplementary Material. Clear-cuttings (for BAU all lands and in CCF, excluding nutrient-rich drained peatlands) were allowed when stand age or mean stand diameter fulfilled the criteria presented in the silvicultural guidelines^40,41^. Stand age and mean stand diameter criteria for stand maturity vary according to the region, site type and tree species.

**Table 5:** Diameter classes of trees (diameter at breast height, 1.3 m, dbh) and harvest intensities applied in the selection harvesting in the CCF scenario.

Diameter class (cm)	Removals when post-harvest basal area is 12–14 m^2^	Removals when post-harvest basal area is 15–17 m^2^
dbh ≥ 27	All removed	All removed
20 < dbh < 27	40% left	60% left
15 < dbh < 20	65% left	70% left
17 < dbh < 15	85% left	85% left

### Estimation of tree biomass carbon stock change for all lands

The forest simulator, MELA estimates tree growth, waste wood (tops of stems and lost logs), natural mortality and harvesting removals for each scenario, by regions and tree species, for 10-year periods (MELA 2021). To estimate the annual carbon sink of trees, stem volume estimates were converted to biomass and carbon. The conversion from stem volume to biomass was conducted using group-specific biomass expansion factors (BEFs) for tree species, which have been developed for the GHG inventory of Finland and vary according to the region and type of biomass (i.e., growth, natural mortality and harvesting), see Appendix 1^42^. These BEFs were estimated from the sample trees of the NFI; for increment BEFs, increment cores and height increment measurements were used for estimating tree biomass and volume (present and 5 years ago), allowing BEF derivation for the increment. For natural mortality and harvesting BEFs, permanent sample data were used, and BEFs were estimated for trees that were either harvested or had died between consecutive inventories. For biomass estimation, empirical models^43,44^ were used, and the model version was chosen according to the availability of the model predictors.

### Estimation of mineral soil carbon exchange

The mineral soil carbon stock change was estimated by using the Yasso07 model^45^. The principles of generating litter inputs and the use of the environmental forcing data and the applied parameters followed those of the GHG inventory of Finland^29^. The main difference between the GHG inventory of Finland and this study was the fact that here, simulations were conducted for each calculation unit separately, not based on regional averages as in the GHG inventory. For details of the application of the Yasso07 model to mineral soils, see the Supplementary Material.

### Estimation of GHG exchange of drained peat soils

To estimate the GHG exchange from drained peatland forests, we applied a stepwise modelling approach. For each calculation unit, we organised data on future scenarios by the MELA model as input to the hydrological SpaFHy-Peat model^10,13,46^ to calculate the daily water table levels. Resulting ditch depths and leaf area indices (LAI) drove water table levels. With the BAU management ditches were deeper, but LAI values were lower, resulting water table levels that were deeper for CCF (see Supplementary Fig. S3 in the Supplementary Material). Thereafter, the annual GHG exchange was estimated based on the mean growing season (mean of May–October) water table levels and site type, coupled with empirical soil GHG exchange models^11,12,30^. We also incorporated a temporary increase in CO_2_ and N_2_O emissions following clear-cut actions and the CH_4_ emissions from ditches. Further, the decomposition and CO_2_ exchange of harvest residues and natural mortality on the oxic layer in drained peatlands were quantified employing the Yasso07 model. Thereafter, the emissions were summed to the regional level by using the emissions and sinks per area and the area represented by each calculation unit. To convert other GHGs to CO_2_ equivalents, global warming potential values of 25 and 298 for CH_4_ and N_2_O, respectively, were applied, based on the IPCC Assessment Report 4^47^. For details on the GHG exchange estimation in drained peatland soils, see the Supplementary Material.

### Supplementary Information

Supplementary Information.

## Data Availability

Plot-level national forest inventory data (NFI11 and NFI12) are available through opendata.luke.fi portal. For tree-level data, please get in touch with the national inventory team of the Natural Resources Institute Finland.

## Code availability

The SpaFHy-Peat soil hydrological model is available through Github: https://github.com/LukeEcomod/SpaFHy-Peat. The Yasso07 soil carbon model is available through Github: https://github.com/YASSOmodel. For the MELA forest simulator, please get in touch with the model developing group of the Natural Resources Institute Finland.
