# Supplementary material for: Potential of continuous cover forestry on drained peatlands to increase the carbon sink in Finland
Source: Sci Rep. 2023 Sep 27;13:15510. doi: 10.1038/s41598-023-42315-7 (PMC10533540; doi:10.1038/s41598-023-42315-7)
Supplement: Supplementary file 1 — Supplementary Information. [file 41598_2023_42315_MOESM1_ESM.pdf]

Supplementary material for Lehtonen et al.: Potential of continuous cover forestry on drained peatlands to increase the carbon sink in Finland?

Aleksi Lehtonen, Kyle Eyvindson, Kari Härkönen, Kersti Leppä, Aura Salmivaara, Mikko Peltoniemi, Olli Salminen, Sakari Sarkkola, Samuli Launiainen, Paavo Ojanen, Minna Räty and Raisa Mäkipää

Segmentation of Finnish forests

Thematic forest map layers of the Finnish multi-source national forest inventory (MS-NFI) from 2015 were used as the basis for automatic forest stand delineation, applying automatic image segmentation based on the "segmentation by directed trees" algorithm[1]. The method employs the local edge gradient in finding potential segment borders[2,3].

Estimating ditch spacing

The segmentation raster was vectorised, and the resulting polygons were converted from singlepart to multipart so that each polygon corresponded to one segment. Forest segments are expected to cover a continuous area, and thus, each polygon should consist of a limited amount of singlepart polygons. In the segmentation process, there were some polygons created that included too many singlepart ones, which were excluded from the analysis. Segment polygons were buffered by 20 m to account for the draining effect of ditches located close to the boundary of the segment. Lines representing the ditch network[4] were overlaid with the buffered segments. The total length of ditches (DL) was extracted for each buffered segment. The hectare-specific ditch length, i.e., ditch density (DD), and ditch area were calculated for each segment using the unbuffered segment area. In the ditch area calculation, the average ditch width of 1 m was assumed, and the overlaps at the joints of ditches were ignored.

As the DD does not directly indicate the actual ditch spacing (DS), we established a link between DD and DS by collecting ditch spacing values calculated for the South Savo region and compared those with the DD values. During the examination, segments smaller than 0.6 ha turned out to require correction to the DD values. These were calculated with Equation S1:

$$DD_{corr} = \frac{DL}{A/c}*25.3, \quad \text{(S1)}$$

where ditch length, DL [m], was divided by the ratio of segment area A [m^2] and segment circumference c [m] and multiplied by 25.3 (a value fitted based on comparing measured DS and DD values). Furthermore, DD (and DD_{corr}) values above 1,200 were replaced by 1,200. Equation S2 was then formulated to convert DD (and DD_{corr}) to DS:

$$DS = DD \times -0.047 + 65.871 \tag{S2}$$

The resulted DS values lower than 20 m were replaced by the value 20 m, and values above 60 m were replaced by the value 60 m.

Altogether, 125 randomly selected segments (25 per 5 regions of Eastern Finland, Western Finland, Northern Ostrobothnia, Lapland, Åland) were checked and classified to classes of a 10-m range (20–30 m, 30–40 m, 40–50 m, 50–60 m). Of these 125 segments, 60% were correctly classified. The average difference was 4.6 m, whereas if one DS value of 40 m was used for all drained segments, the difference would be 11.2 m. This suggests that using the estimated DS improves the accuracy compared to using one average ditch spacing value for all drained segments.

Ditch depth estimation

The maintenance ditching operation was always conducted with the MELA simulations after clear-cut on drained peatland forests. Maintenance ditching was conducted during thinnings on poorer sites and on more nutrient-rich sites in the BAU scenario if that was found profitable. The impact maintenance ditching to ditch depth was also included in the SpaFHy-Peat simulations if that was indicated by the MELA output.

To estimate initial ditch depth by region and site type, NFI12 data from 2014–2018 were analysed for ditch property information, and predictions were made with a ditch depth model[5] (Table S1). To estimate ditch depth during MELA simulations, the type of ditching activity and time of ditching were used from the MELA data with the ditch depth model. For the model application, also the north coordinate and peat layer depth values up to 120 cm of each NFI plot were provided to obtain ditch depth estimates for each calculation unit with ditches. Thereafter, the development of ditch depths followed the model assuming an average ditch depth of 60 cm, with a minor slope of less than half a percentage gradient, being 0.46.

Table S1. Mean and median ditch depth estimates based on the NFI12 data and ditch depth models[5].

Region	Count	Mean	Median	Variance
Åland	7	39.3	41.1	19.5
Central Finland	687	50.1	43.4	275.9
North Karelia	972	49.6	41.8	286.3
South Savo	550	50.2	42.9	271.2
Southern Ostrobothnia	856	57.7	57.9	307.3
Ostrobothnia	329	55.8	49.7	273.7
North Savo	689	52.6	44.1	315.9
Central Ostrobothnia	381	55.3	46.7	349.1
Northern Ostrobothnia	2,189	52.0	42.6	306.0
Kainuu	1,256	49.4	41.8	261.5
Uusimaa	127	47.1	41.4	291.9
Southwest Finland	211	50.5	44.6	307.2
Kymenlaakso	124	51.6	41.1	351.6
Kanta-Hame	155	48.9	42.6	265.3
Päijät-Hame	98	48.2	41.1	299.1
South Karelia	187	51.9	42.6	374.0
Satakunta	417	53.8	49.7	301.3
Pirkanmaa	421	51.3	46.1	280.1
Lapland	1,014	44.3	40.0	138.0

Weather forcing for SpaFHy-Peat

Weather forcing information was extracted from gridded weather predictions (10 × 10 km^2) by the Finnish Meteorological Institute[6]. Water table depth predictions with SpaFHy-Peat require daily temperature, rainfall, global radiation and vapor pressure deficit as the input. Each calculation unit was simulated for each of the 16 years of weather data from 2000–2015 and future weather data for the simulation period were derived by repeating the historical weather pattern data of the period 2000–2015.

Leaf area index

The estimation of the leaf area index (LAI) of trees followed the methodology in which biomass estimates are converted to LAI with specific leaf area factors[7]. Here, foliage biomass was estimated using models[8,9] at tree level, based on the calculation unit data from MELA simulations, and the data were converted to one-sided LAI by using species-specific leaf area-to-biomass ratios and according to canopy openness (the fraction of unobscured sky). For understory vegetation, the development of the LAI was estimated as function stand basal area (Table S2).

Table S2. LAI [m^2 m^{-2}] estimation for understorey vegetation as function stand basal area (G).

Stand min G	Stand max G	LAI estimate for understorey vegetation
0	0.49	0.5
0.5	4.9	1.5
5	29.9	$-0.056 \times G + 1.78$
30		0.3

Estimation of the water table level

To estimate the daily water table level for each calculation unit, we applied the SpaFHy-peat model. The model integrates descriptions of aboveground hydrology from SpaFHy with a simple hydrological description of the peat profile accounting for soil water storage and lateral ditch drainage to obtain the daily water table level[10]. The model runs on daily meteorological forcing, and its key drivers for water table level are leaf area index (LAI), peat type (*Sphagnum* peat or *Carex* peat), ditch spacing and ditch depth. The MELA output on leaf biomass, converted to tree leaf area index development, harvestings, soil type, site type fertility, ditching and calculation unit location, was used as input for SpaFHy-Peat.

Application of GHG exchange models for drained peatland soils

Stands with tree cover

For each calculation unit with tree cover (excluding recently clear-cut areas), annual soil CO_2, CH_4 and N_2O emissions and sinks were estimated by applying GHG estimation models with site mean growing season water table level and site fertility as predictors, as presented elsewhere[11–13].

CH_4 emissions from ditches

In addition to soil emissions described above, we estimated CH_4 emissions from ditches. Methane emissions from ditches were approximated based on empirical observations collected during the N_2O emission study, using similar methods[11]. The annual CH_4 emissions (e_{ch4}) from ditches were estimated by ditch depth (d) in cm, using the following equation:

$$e_{ch4} = \frac{d}{(a_0 + a_1 \times d)}, \quad \text{(S4)}$$

where and a_0 and a_1 are parameters (Table S3). The resulting emission estimates of CH_4 are presented in kg ha^{-1}.

Table S3. Parameter estimates for ditch CH_4 emissions [kg CH_4 m^{-2} year^{-1}].

Parameter, CH_4 ditch	a_0	a_1
	3.32	0.0095

Emissions from clear-cut areas

Drained peatlands emit substantial amounts of N_2O and CO_2 during the years following clear-cut[14–16]. To account for these additional clear-cut-induced emissions, we applied a rough approximation of the CO_2 and N_2O emissions for the years after clear-cut.

To consider increased CO_2 emissions since clear-cut, linear models were estimated and applied to the first 9 years after the clear-cut. The equation is as follows:

$$e_{co2} = b_0 + b_1 \times t, \quad \text{(S5)}$$

where e_{co2} is the estimated emission (g CO_2 m^{-2} yr^{-1}), b_0 and b_1 are parameters, and t denotes the time since clear-cut (Table S4). Parametrisation was based on the previous works[14–16]. Following the 9-year period since clear-cut, CO_2 emissions were estimated according to the water table level and emission models[12], as described above.

Table S4. Parameter estimates for CO_2 emissions after clear-cut for the first 9 years [g CO_2 m^{-2} $year^{-1}$].

Parameter, CO_2 clear-cut	b_0	b_1
Nutrient-rich soils	2,655.9	-276.5
Nutrient-poor soils	2,000	-200

For N_2O emissions after clear-cut, for nutrient-rich sites, we used a model with linear decrease from 4 to 1 g m^{-2} for the first 10 years[15]. For nutrient-poor sites (*Vaccinium* type and less fertile), we used a similar linear decrease from 1 to 0.2 g m^{-2} for the first 5 years, based on expert judgement. For N_2O emissions (e_{n2o}) from nutrient-rich and nutrient-poor sites, we used the following equation:

$$e_{n2o} = b_0 + b_1 \times t, \quad \text{(S6)}$$

where *y* is the time since clear-cut, and b_0 and b_1 are parameters (Table S5). Following the 10-year (nutrient-rich sites) or 5-year period (nutrient poor sites) since clear-cut, N_2O emissions were estimated according to the water table level and emission models[11], as described above.

Table S5. Parameter estimates for N_2O emissions after clear-cut for the first 10 and 5 years for nutrient-rich and nutrient-poor sites, respectively [g CO_2 m^{-2} $year^{-1}$].

Parameter, N_2O clear-cut	b_0	b_1
Nutrient-rich soils	4	-0.3
Nutrient-poor soils	1	-0.2

Application of the Yasso07 soil carbon model for carbon exchange of mineral soils

The initialisation of the Yasso07 soil carbon model[17] was conducted by each calculation unit of MELA. Spatially nearest daily weather data, based on the observations from weather stations, were obtained from the gridded (10 x 10 km) weather database produced by the Finnish Meteorological Institute[6]. The Yasso07 soil model was run from 2000 onwards, with initial carbon stocks reflecting measured averages[18], following simulation with component-specific litter fall estimates (i.e., foliage, branches, stem and roots) from 2000 to NFI data start (measurements done in 2012–2018). Thereafter, each initial calculation unit data (measured NFI plot) was back-casted with an annual reduction of 2.5% to derive the historical litter fall estimates between the year 2000 and the year of NFI measurement (i.e., start of MELA simulations). The assumed 2.5% annual reduction was based on expert judgement to generate appropriate initial carbon stocks for the Yasso07 model. In the case of recently clear-cut calculation units without any tree biomass, the mean litter input estimate of the future prediction was used to estimate historical litter fall and resulting spin up soil C stocks. Thereafter, annual litter fall estimates were generated from the MELA outputs for each calculation unit for future scenarios. Annual average weather data (mean temperature, temperature amplitude and rainfall) from 30 years before the measurement, localised for each calculation unit, were used for the spin-up 2000 to the NFI data start. For estimating future soil carbon stock changes, weather data from 2016 were used for each location from 2016 onwards.

Regional data and results

Tables S6, S7 and S8 provide land areas of Norway spruce-dominated nutrient-rich drained peatlands with mature stands, and drained peatlands and undrained peatland forests in Finland by site type and region.

Most of the emissions reductions were attributed for those regions that had the largest areas with nutrient-rich drained peatlands and areas that have high harvesting possibilities. Larger climate benefits with CCF compared with BAU were also found for Pirkanmaa, which can be attributed to higher tree biomass sink under CCF management. In such stands, conversion to CCF resulted in an extension of the rotation period of the stands, where, instead of clear-cuttings, additional selection harvestings were conducted. The higher GHG sink associated with CCF for Kymenlaakso can be attributed to the fact that harvestings were approximately 100,000 m^3 (4.4%) higher for BAU than for CCF during the 2nd and 3rd simulation periods, as the MELA simulator did not find enough

timber to be harvested under CCF scenario. This is related to the fact that in Kymenlaakso actual harvestings have been higher than the maximum sustained yield since 2016[19], and conversion from BAU to CCF would not provide equally large harvesting amounts from this region. Typically, in the regions where the conversion to CCF increased the tree biomass and reduced the soil GHG-emissions, the area of mature tree stands on nutrient-rich drained peatlands was larger compared with other regions.

Table S6. Land areas of Norway spruce-dominated nutrient-rich drained peatlands with mature stands (development classes of advanced thinning stand and mature stand) in Finland [1,000 ha] according to NFI12/13 data (2017-2021) on forest land. Regions with more than 25,000 ha of nutrient-rich drained peatland identified with bold.

Region	Eutrophic fens	Herb-rich fens	Tall-sedge fens and *Vaccinium myrtillus* swamps	Total
Uusimaa		4.5	3.1	7.6
Southwest Finland		7	11.4	18.4
**Satakunta**	**0.3**	**12.3**	**17.4**	**30.1**
Kanta-Häme	0.7	7.3	10.7	18.7
**Pirkanmaa**		**11.3**	**17.5**	**28.8**
Päijät-Häme		5.7	4.7	10.3
Kymenlaakso		2.4	4.2	6.6
South Karelia	0.3	1.6	3.9	5.8
South Savo	1	7.9	14.1	23
**North Savo**	**0.4**	**11.6**	**19.9**	**32**
**North Karelia**	**0.8**	**6.5**	**29.8**	**37.2**
**Central Finland**		**7.8**	**21.7**	**29.5**
Southern Ostrobothnia	0.8	3.7	16.3	20.8
Ostrobothnia	0.4	7	12.9	20.3
Central Ostrobothnia		1.7	3.8	5.4
**Northern Ostrobothnia**	**1.4**	**10.6**	**32.6**	**44.5**
Kainuu	0.5	7.3	11	18.7
**Lapland**		**7.9**	**25.2**	**33.1**
Åland		0		0
Total Finland	6.6	124.1	260.2	390.9

Table S7. Areas of drained peatlands in Finland [1,000 ha] according to National Forest Inventory data measured in 2017–2021 on productive and poorly productive forests.

Region	Eutrophic fens	Herb-rich fens	Tall-sedge fens and *Vaccinium myrtillus* swamps	Low-sedge fens and *V. vitis-idaea* swamps	Cotton grass and Dwarf-shrub bogs	*Sphagnum fuscum* bogs	Total
Uusimaa	1	15	12	6	5	-	39
Southwest Finland	1	19	34	14	14	2	84
Satakunta	1	30	53	40	20	2	146
Kanta-Häme	2	16	26	12	5	1	62
Pirkanmaa	3	35	60	59	26	2	185
Päijät-Häme	1	11	17	8	0	-	38
Kymenlaakso	2	6	19	12	5	-	44
South Karelia	4	10	25	15	12	0	66
South Savo	7	27	70	46	20	0	171
North Savo	12	46	98	75	45	2	279
North Karelia	12	33	138	112	102	5	403
Central Finland	2	31	95	91	51	4	273
Southern Ostrobothnia	4	17	79	155	97	11	362
Ostrobothnia	2	15	50	32	27	2	127
Central Ostrobothnia	-	4	29	44	59	5	142
Northern Ostrobothnia	6	73	282	454	243	18	1,076
Kainuu	3	37	102	242	131	4	518
Lapland	2	53	153	358	177	2	745
Åland	0	0	0	0	0	-	2
Total Finland	64	479	1,342	1,774	1,040	60	4,759

Table S8. Areas of undrained peatlands in Finland [1,000 ha] according to National Forest Inventory data measured in 2017–2021 on productive and poorly productive forests.

Region	Hardwood-spruce mires	Pine mires	Total
Uusimaa	12	6	18
Southwest Finland	11	19	30
Satakunta	15	16	31
Kanta-Häme	6	7	14
Pirkanmaa	16	18	34
Päijät-Häme	7	3	10
Kymenlaakso	8	5	13
South Karelia	11	2	13
South Savo	20	17	37
North Savo	35	25	60
North Karelia	37	59	95
Central Finland	27	32	59
omaSouthern Ostrobothnia	18	45	62
Ostrobothnia	17	13	31
Central Ostrobothnia	4	18	22
Northern Ostrobothnia	83	265	347
Kainuu	63	124	187
Lapland	306	770	1,076
Åland	2	3	5
Total Finland	698	1,447	2,145

Table S9. Differences in GHG exchange [Gg CO_2 eq.] of regions between the CCF and BAU scenario at actual harvesting levels for 2022–2035 and 2022-2050. On the left for the total GHG exchange for ecosystems, on the right for the soil GHG exchange. Positive values in the table indicate that the CCF scenario has a less favorable impact on GHG emissions than the BAU scenario.

**Component**	**Total ecosystem GHG exchange difference (CCF-BAU)**		**Soil GHG exchange difference (CCF-BAU)**	
**Region**	**2022–2035**	**2022–2050**	**2022–2035**	**2022–2050**
Uusimaa	-47.3	-83.6	-67.3	-84.1
Southwest Finland	-78	-20.6	-65	-11.6
Satakunta	-216.2	-6	-164.4	3.6
Kanta-Häme	-82	-33.2	-19.1	8.9
Pirkanmaa	-119	-110.8	-28.7	-30.8
Päijät-Häme	19.1	96.2	-10.1	-28.2
Kymenlaakso	-107.3	-142.8	2.5	24.8
South Karelia	-41	-53.6	-25.5	-26.2
South Savo	35.4	34.6	-14.6	-24.3
North Savo	-81	-39.4	-141.2	-103.2
North Karelia	-113.3	-108.1	-77.6	-33.1
Central Finland	-130.2	-137.4	-109.6	-96.8
Southern Ostrobothnia	-64.9	-44.2	-47.2	-10.6
Ostrobothnia	-33.8	-64.3	-84	-86.2
Central Ostrobothnia	-45.3	-44.1	-25.1	-22.9
Northern Ostrobothnia	-1.3	-91.1	71.1	46
Kainuu	-33.4	-25.3	-6.4	3.8
Lapland	-136.7	-70.5	-88.7	-32.5
Åland	-7.5	-4	-3.3	-2.8
**Total**	**-1,283.7**	**-948.2**	**-903.9**	**-506.1**

Other supplementary information

The sensitivity analysis based on the assumption where the growth of suppressed Norway spruce trees is reduced by 25% for first 5 years (CCF-reg) after selection harvests have been provided in the Table S10. Figure S1 presents applied thinning rules with the MELA model, in the case of

selection harvest, while Figure S2 presents the impacts of assumptions with clear-cut related CO_2 and N_2O emissions.

Table S10. GHG exchange [Tg CO_2 eq.] of Finland according to the CCF-reg scenario at actual harvesting (AH) and maximum sustained yield (MSY) levels for Finland.

Harvestings		2022–2027	2028–2037	2038–2047
Actual harvestings	Tree biomass	-14.95	-16.69	-20.05
Maximum sustained yield	Tree biomass	-8.79	-1.41	-0.83
Actual harvestings	Organic soils	12.9	12.57	12.41
Maximum sustained yield	Organic soils	11.15	11.13	11.5
Actual harvestings	Mineral soils	-7.1	-7.43	-11.03
Maximum sustained yield	Mineral soils	-2.68	-3.93	-6.26
Actual harvestings	Total	-9.16	-11.55	-18.67
Maximum sustained yield	Total	-0.32	5.78	4.4

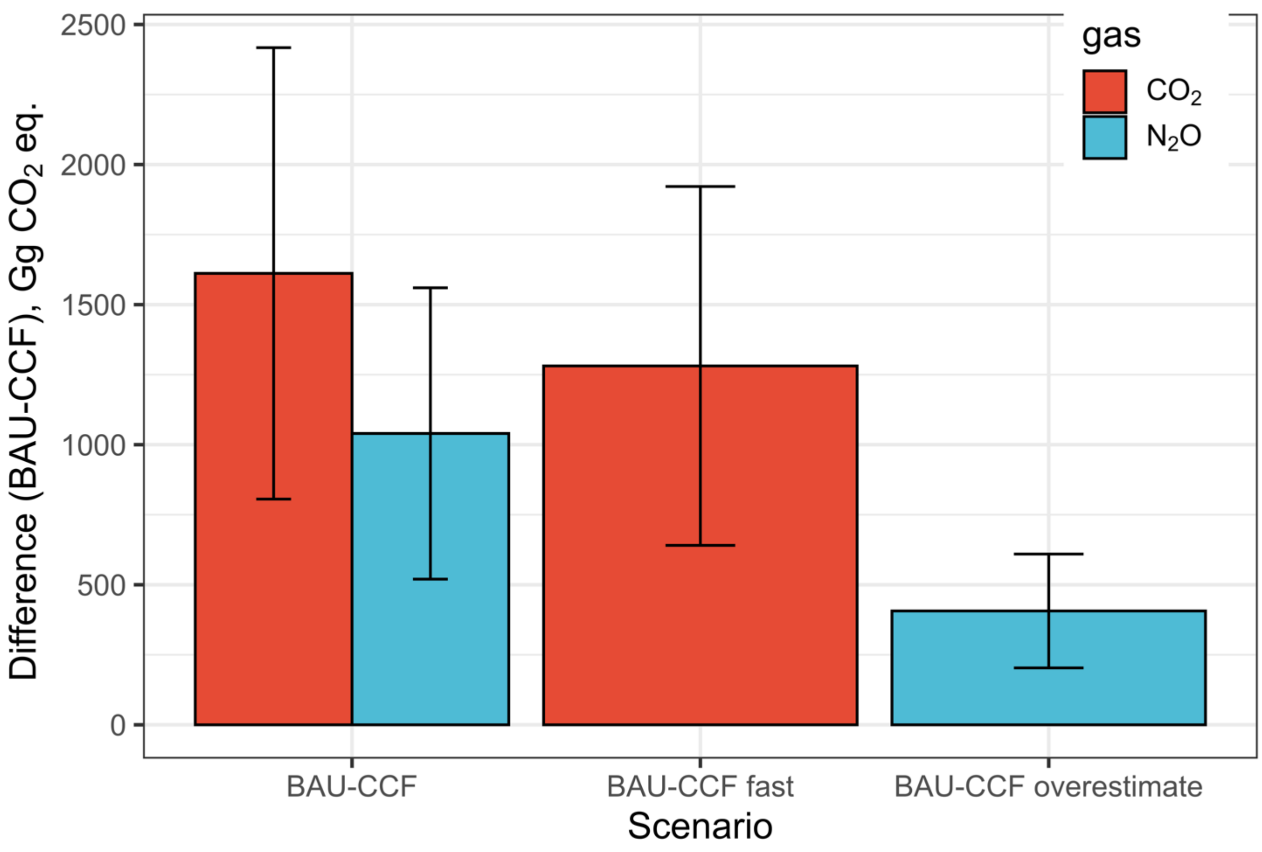

Figure S1. Impacts of clear-cut areas and used emission factors on the difference between the BAU and CCF scenarios, with 50% standard deviation in the emission factors, using MELA estimates for clear-cut areas for drained peatlands. Note: nutrient-rich drained peatlands in square brackets (74,500 [29,690] ha and 60,900 [13,940] ha, respectively, for BAU and CCF). The graph shows the difference between emissions with BAU and CCF after 9 years of implementing clear-cuts of given areas on drained peatland forests. BAU-CCF indicates differences when using the time-dependent emission factors described in the paper, BAU-CCF fast indicates the case where CO_2 reduces over 8 years, instead of assumed 10 years after clear-cut, and BAU-CCF overestimate indicates the case where N_2O emissions start from 2 g per m^2 per year and decrease by -0.2 g per m^2 per year after clear-cut.

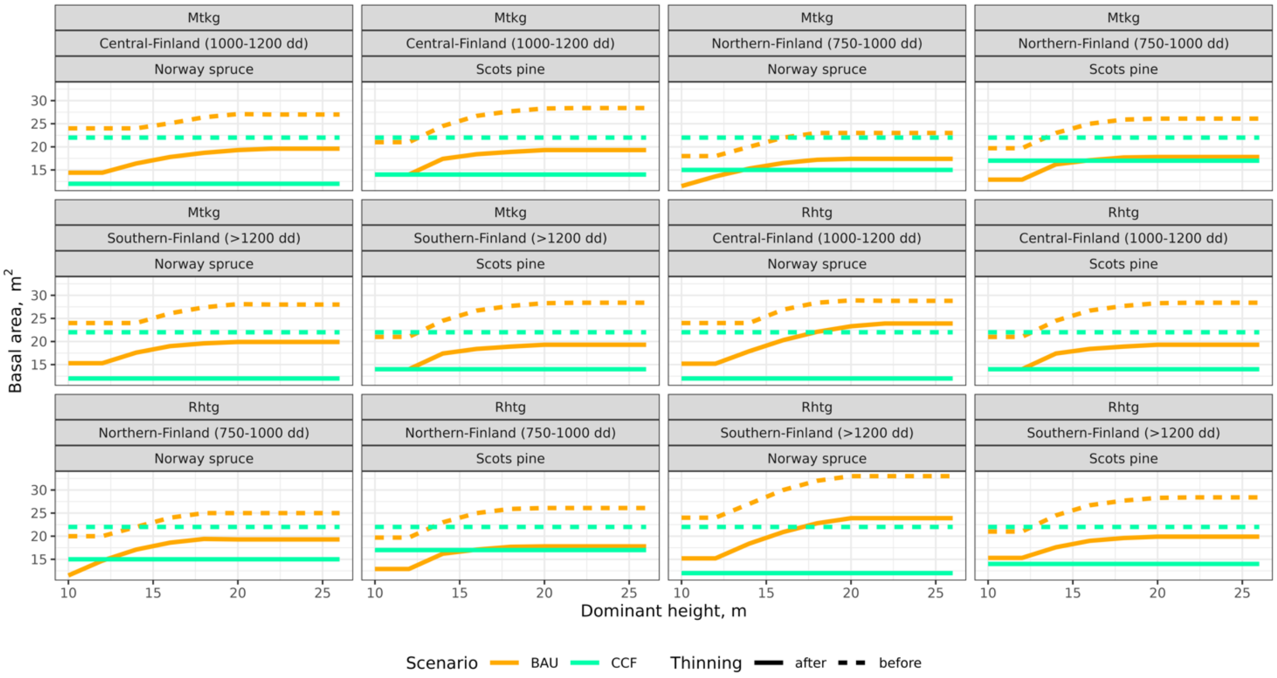

Figure S2. Applied thinning rules with the MELA simulator for Scots pine- and Norway spruce-dominated stands for BAU (orange) and for CCF (green). Dashed lines indicate basal area levels before thinning, and solid lines indicate those after thinning. Basal area [m^2] thresholds shown as a function of dominant height [m] of the stand. Mtkg is the *Vaccinium myrtillus* type site, and Rhtg is the most nutrient-rich herb-rich type site according to the Finnish site type classification system of forestry drained peatlands.

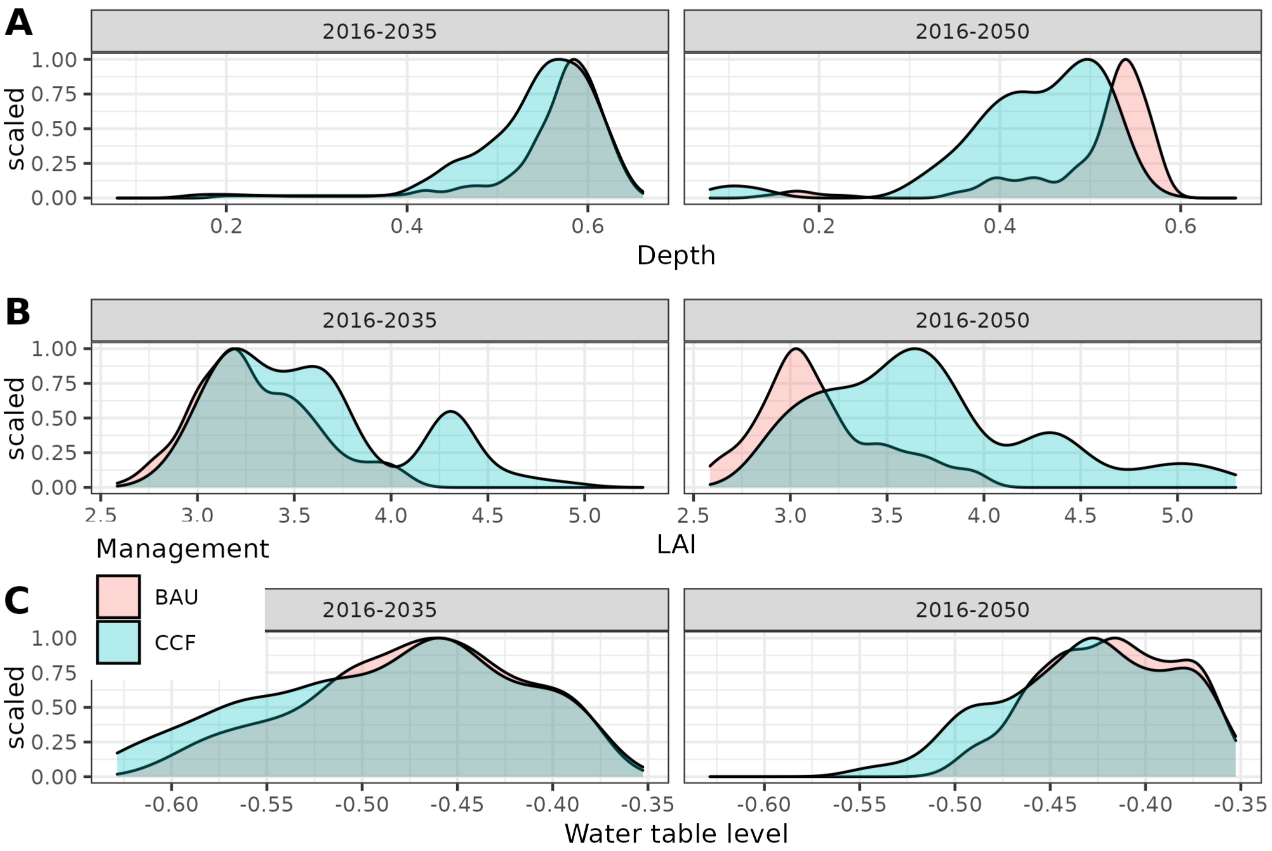

Figure S3. Distributions as kernel density estimates of ditch depth (A) [m], leaf area index (B) [m^2 m^{-2}] and resulting water table level (C) [m] for BAU and CCF management under actual harvesting scenario for 2016-2035 and 2016-2050 for all drained peatland sites in Finland.

References

1. Narendra, P. M. & Goldberg, M. Image Segmentation with Directed Trees. *IEEE Transactions on Pattern Analysis and Machine Intelligence* **PAMI-2**, 185–191 (1980).
2. Pekkarinen, A. Image segment-based spectral features in the estimation of timber volume. *Remote Sensing of Environment* **82**, 349–359 (2002).
3. Haakana, M., Tuominen, S., Heikkinen, J., Peltoniemi, M. & Lehtonen, A. Spatial patterns of biomass change across Finland in 2009–2015. *ISPRS Open Journal of Photogrammetry and Remote Sensing* **8**, 100036 (2023).
4. National Land Survey of Finland (NLS). Streams and ditches. The Topographic Database. (2019).
5. Hökkä, H., Stenberg, L. & Laurén, A. Modeling depth of drainage ditches in forested peatlands in Finland. *BALT FOR* **26**, (2020).
6. Venäläinen, A., Tuomenvirta, H., Pirinen, P. & Drebs, A. *A basic Finnish climate data set 1961-2000 – Description and illustrations*. 27 (2005).
7. Härkönen, S., Lehtonen, A., Manninen, T., Tuominen, S. & Peltoniemi, M. Estimating forest leaf area index using satellite images: comparison of k-NN based Landsat-NFI LAI with MODIS- RSR based LAI product for Finland. *Boreal Environment Research* **20**, 15 (2015).
8. Repola, J. Biomass equations for Scots pine and Norway spruce in Finland. *Silva Fennica* **43**, 625–647 (2009).
9. Repola, J. Biomass equations for birch in Finland. *Silva Fennica* **42**, 605–624 (2008).
10. Leppä, K. *et al.* Selection Cuttings as a Tool to Control Water Table Level in Boreal Drained Peatland Forests. *Front. Earth Sci.* **8**, 576510 (2020).

11. Minkkinen, K., Ojanen, P., Koskinen, M. & Penttilä, T. Nitrous oxide emissions of undrained, forestry-drained, and rewetted boreal peatlands. *Forest Ecology and Management* **478**, 118494 (2020).

12. Ojanen, P. & Minkkinen, K. The dependence of net soil CO2 emissions on water table depth in boreal peatlands drained for forestry. *Mires and Peat* 1–8 (2019) doi:10.19189/MaP.2019.OMB.StA.1751.

13. Ojanen, P., Minkkinen, K., Alm, J. & Penttilä, T. Soil–atmosphere CO2, CH4 and N2O fluxes in boreal forestry-drained peatlands. *Forest Ecology and Management* **260**, 411–421 (2010).

14. Mäkiranta, P., Riutta, T., Penttilä, T. & Minkkinen, K. Dynamics of net ecosystem CO2 exchange and heterotrophic soil respiration following clearfelling in a drained peatland forest. *Agricultural and Forest Meteorology* **150**, 1585–1596 (2010).

15. Korkiakoski, M. *et al.* Partial cutting of a boreal nutrient-rich peatland forest causes radically less short-term on-site CO2 emissions than clear-cutting. *Agricultural and Forest Meteorology* **332**, 109361 (2023).

16. Korkiakoski, M. The short-term effect of partial harvesting and clearcutting on greenhouse gas fluxes and evapotranspiration in a nutrient-rich peatland forest. (Finnish Meteorological Institute, 2020). doi:10.35614/isbn.9789523361300.

17. Tuomi, M., Rasinmäki, J., Repo, A., Vanhala, P. & Liski, J. Soil carbon model Yasso07 graphical user interface. *Environmental Modelling & Software* **26**, 1358–1362 (2011).

18. Peltoniemi, M., Mäkipää, R., Liski, J. & Tamminen, P. Changes in soil carbon with stand age – an evaluation of a modeling method with empirical data. *Global Change Biology* **10**, 2078–2091 (2004).

19. Vaahtera, E. *et al.* Metsätilastollinen vuosikirja 2021. (2021).
